# Regional level influenza study based on Twitter and machine learning method

**DOI:** 10.1371/journal.pone.0215600

**Published:** 2019-04-23

**Authors:** Hongxin Xue, Yanping Bai, Hongping Hu, Haijian Liang

**Affiliations:** 1 School of Information and Communication Engineering, North University of China, Taiyuan, Shanxi, 030051, People’s Republic of China; 2 Department of Mathematics, School of Science, North University of China, Taiyuan, Shanxi, 030051, People’s Republic of China; 3 National Key Laboratory for Electronic Measurement Technology, Key Laboratory of Instrumentation Science & Dynamic Measurement Ministry of Educations, School of Information and Communication Engineering, North University of China, Taiyuan, Shanxi, 030051, People’s Republic of China; Universitatsmedizin Greifswald, GERMANY

## Abstract

The significance of flu prediction is that the appropriate preventive and control measures can be taken by relevant departments after assessing predicted data; thus, morbidity and mortality can be reduced. In this paper, three flu prediction models, based on twitter and US Centers for Disease Control’s (CDC’s) Influenza-Like Illness (ILI) data, are proposed (models 1-3) to verify the factors that affect the spread of the flu. In this work, an Improved Particle Swarm Optimization algorithm to optimize the parameters of Support Vector Regression (IPSO-SVR) was proposed. The IPSO-SVR was trained by the independent and dependent variables of the three models (models 1-3) as input and output. The trained IPSO-SVR method was used to predict the regional unweighted percentage ILI (%ILI) events in the US. The prediction results of each model are analyzed and compared. The results show that the IPSO-SVR method (model 3) demonstrates excellent performance in real-time prediction of ILIs, and further highlights the benefits of using real-time twitter data, thus providing an effective means for the prevention and control of flu.

## Introduction

Influenza (flu) is a stealthy killer that threatens human health with its widespread contagion [1, 2]. The flu refers to a viral acute respiratory infection caused by the common flu virus. If the flu is not effectively controlled, it can cause wide-ranging flu outbreaks that pose a threat to social stability and development. The World Health Organization (WHO) asserts that about 3 to 5 million serious illnesses are reported worldwide each year and about 250,000-650,000 of those result in death [3]. If we can predict a flu trend in some areas before the outbreak of flu, and take effective measures to mitigate the contagion ahead of time, we can control the spread of disease and reduce the loss of life to a certain extent.

To prevent and control flu pandemic, the current worldwide Flu Surveillance System (FSS) relies on the collaboration of medical institutions (at all levels), e.g., centers for disease control and prevention and sentinel hospitals. The worldwide FSS monitors the flu weekly, via accurate reporting, and releases the information to regional centers [4]. In the US, the CDC releases weekly health data at the national and state level to determine when and where flu outbreak reaches the US to measure the impact of the epidemic on the whole country [5].

With the development of the information technology field, new efficient data sources are continuously produced by a variety of reporting agencies. In the US, about 90 million adults search for health information [6], such as disease and medicine, on the internet every year. When a flu outbreak occurs, people often learn about the outbreak (and how to deal with it) via search engines like Baidu or Google [7, 8]. Therefore, internet search data has become an ideal data source for flu surveillance [9–12]. Google analyzed the data from its own search engine and found that there was a relationship between the number of people who searched for flu-related subjects and the number of people who had flu symptoms [13]. In 2008, Google launched the Google Flu Trend (GFT), based on aggregated Google search data that estimated the current global flu transmission in near real time [14, 15]. Although some success was achieved, a February 2013 *Nature* magazine article pointed out that, compared with data from CDC, the GFT overestimated the peak number of ILI in the US [16]. Millions of engineers and users are constantly changing search engine algorithms [17–22], but ultimately, the GFT was shut down in August 2015.

Many other data sources are actively searching for a precise way to perform correlation analysis with flu data [23, 24], particularly by companies (sources) who sell over-the-counter (OTC) medication that reduce the symptoms of the flu such as fever, body ache, coughing and sneezing [25]. Much of the literature on disease surveillance using social media has focused on tracking influenza with twitter [26–28]. Twitter is a popular social network. There are over 600 million users as of January 2014, generating over 780 million tweets daily [29]. Twitter data is appealing as a data source because the application can access millions of public short messages instantly every day. The twitter application has become a viable option for disseminating and tracking information. Although twitter, as a social network, appears to be targeted to a young generation, the demographic breakdown of the social network reveals that users of the twitter application are diverse in terms of age. The social network is not only for young people, but also for middle aged and the technology savvy older population [30].

Previous work has drawn upon novel web data-based twitter application messaging models to detect influenza rates in real time, to infer health status or measure the spread of a disease in a population. For instance, Paul et al. [31] uses ILI data available at the time of the forecast show that models incorporating data derived from twitter can reduce forecasting error. Kim et al. [32] proposed an adaptive algorithm for real-time prediction of influenza infection and actual disease activity using the Hangeul Twitter. Hu et al. [33] proposed an improved artificial tree algorithm to optimize the parameters of BP neural networks (IAT-BPNN) that can predict the CDC’s %ILI of US. Signorini et al. [34] applied content analysis and regression models to measure and monitor public concern about the levels of disease during the H1N1 pandemic in the US. The CDC used the twitter application to post tips for preventing flu to help slow the spread of H1N1 influenza in 2009. The twitter’s account grew from 2,500 followers to 370,000 followers during the 2009 outbreak [35]. Lampos [36] analyzed twitter messages using regression models in the UK and the US, respectively. Broniatowski et al. [37] argued that the twitter social network produces an open data collection and the interests in flu and the number of real flu cases are separable in twitter flu data.

The biggest advantage of these methods, compared to traditional methods, is the immediate feedback: Twitter message and/or query log analyses are available almost immediately. This is extremely important to prevent influenza, as early detection can reduce the impact of flu outbreaks. Although many studies targeting flu prediction using twitter data have been presented, most of these methods simply use linear regression algorithm to predict %ILI. These methods do not consider geographical to their models, for example, they do not consider regional correlation in their flu-spread model. In this study, we improve the short-term predictions of flu activity by using inter-regional ILI correlation, and propose a non-linear methodology based on machine learning algorithms capable of providing real-time (“nowcast”) and forecast estimates of %ILI by leveraging twitter application and CDC data.

Machine learning (ML) is a type of artificial intelligence method that has reemerged to analyze large data typically called “big data.” ML applications are becoming increasingly widespread as the amount of available information increases exponentially. For example, we can apply ML algorithms to improve hospital-based expert systems [38], bibliographic classification [39, 40], automatic target tracking algorithms [41], the implementation of computer-based GO games [42] and the optimization of driverless car algorithms [43]. In fact, we can apply ML methods to model almost any aspect of human life so that we may develop innovative technological tools that can improve living conditions.

SVR is a type of ML method developed from pattern recognition and computational learning that stems from statistics; it is a theoretical tool with excellent performance that can 1) guarantee the global optimum of an algorithm and 2) adopt a kernel function method that avoids complex operations and solves the problem of high-dimensionality [44]. In the present study, we propose an improved PSO to optimize the parameters of SVR. The independent and dependent variables of models 1-3 are used as input and output of IPSO-SVR for predicting the CDC’s unweighted %ILI of US. The aim of the present study is to evaluate the application of this ML approach applied to flu prediction. Although these models have not been used for national or regional %ILI predictions in the past, they can be reference models against which new methods can be tested.

Important novelty of our work is: the impact of flu transmission between geographical regions are analyzed and verifies whether the CDC ILI are complementary to the twitter data; we develop a correction to the existing PSO algorithm that optimizes a penalty parameter *C* and kernel function parameter *σ* of an SVR-based model that improves the prediction for %ILI. The resulting model, in turn, can be employed to forecast influenza epidemics in the US, which may help to facilitate vaccination-strategy development and antiviral distribution.

## Models

Historical twitter data mapped onto ILI contains a lot of information about flu epidemic from previous years, which has important significance for future flu trend-based predictions. Therefore, we develop model 1 by historical twitter data on ILI. The flu is an acute infectious disease with the ability to spread in physical space. Population regions that are geographically near each other will likely experience highly correlated patterns of flu cases. Therefore, we construct an empirical network model (model 2) using twitter data to verify the regional impact of flu transmission. In traditional flu prediction model development, the data becomes more accurate after rigorous scientific experimentation. Various forecasting methods have their own advantages and disadvantages. Therefore, we construct a combination model (model 3) by introducing CDC ILI data to model 2. Model 3 verifies whether the twitter data is complementary to CDC ILI data. Model 3 also determines whether the twitter data contains new information that is not provided by the historical CDC ILI data. The specific formulas of models 1-3 are

Model 1:
  $$\begin{array}{c} ILI_{i,t}=\sum_{k=1}^{p}\alpha_{k}X_{i,t-k}+\varepsilon_{t}. \end{array}$$ (1)
Model 2:
  $$\begin{array}{c} ILI_{i,t}=\sum_{k=1}^{p}\beta_{k}X_{i,t-k}+\sum_{j\neq i,j=1}^{N}\delta_{j}\omega_{i,j}X_{j,t}+\theta_{t}. \end{array}$$ (2)
Model 3:
  $$\begin{array}{c} ILI_{i,t}=\sum_{k=1}^{p}\gamma_{k}X_{i,t-k}+\sum_{l=1}^{q}\mu_{l}ILI_{i,t-l}+\sum_{j\neq i,j=1}^{N}\sigma_{j}\omega_{i,j}X_{j,t}+\tau_{t}. \end{array}$$ (3)

In all models, the *X*_*i*,*t*−*k*_ represents twitter data in the *i*-th region for week *t* − *k*, *ω*_*i*,*j*_ is weighting factor that establishes the relationship between regions *i* and *j* the correlation coefficient of the CDC ILI data in region *i* and *j* represents the relationship weight. *ILI*_*i*,*t*−*l*_ characterizes the CDC ILI data of the *i*-th region for the last *l* weeks, *p*, *q* are the lagged order coefficients (the experimental results show that the prediction effect of the model is best when *p* = *q* = 3) and the coefficients *α*_*k*_, *β*_*k*_, *δ*_*j*_, *γ*_*k*_, *μ*_*l*_ and *σ*_*j*_ are the parameters of the model. The variables *ε*_*t*_, *θ*_*t*_ and *τ*_*t*_ are the residual terms for each model, while *N* is the number of regions (in this case *N* = 10).

## Methods

### Support vector regression

Support Vector Machine (SVM) was first proposed by Vapnik et al. in 1995 [45], and is a ML method based on Vapnik-Chervonenkis (VC) dimension theory and the principle of structural risk minimization. It was first applied to classical classification problems and showing promise in solving nonlinear and high dimensional problems, then the method was applied to common regression problems [46, 47]. An SVM applied to nonlinear regression is called Support Vector Regression (SVR). In this paper, the *ε*-SVR methodology is adopted and its specific form is as follows [48, 49]:

We are given training data

$$\begin{array}{c} S=\{\left(x_{1},y_{1}\right),\left(x_{2},y_{2}\right),\cdots ,\left(x_{k},y_{k}\right)|x_{i}\in R^{n},y_{i}\in R\}. \end{array}$$ (4)

where *x*_*i*_ ∈ *R*^*n*^ is the input vector, *y*_*i*_ ∈ *R* is the corresponding output and *k* is the sample size.

The nonlinear SVR maps the input (data) vectors into a high-dimensional feature space *H* via a nonlinear mapping Φ and then performs linear regression in this space. The specific function can be expressed as

$$\begin{array}{c} f\left(x\right)=\omega^{T}\cdot \Phi \left(x\right)+b,\Phi :R^{n}\to H,\omega \to R^{n}. \end{array}$$ (5)

where *ω* is the weight vector of the hyperplane and *b* is the bias term.

In fact, SVR solves optimization problem (6) under constraint conditions (7).

$$\begin{array}{c} \min_{\omega ,b,\xi_{i},\xi_{i}^{*}}\frac{1}{2}\omega^{T}\omega +C\sum_{i=1}^{k}\left(\xi_{i}+\xi_{i}^{*}\right). \end{array}$$ (6)

with constraint

$$\begin{array}{c} \left\{ \begin{array}{cc} & y_{i}-\left[\omega^{T}\cdot \Phi \left(x\right)+b\right]\leq \varepsilon +\xi_{i}\\ & \left[\omega^{T}\cdot \Phi \left(x\right)+b\right]-y_{i}\leq \varepsilon +\xi_{i}{}^{*}\\ & \xi_{i},\xi_{i}{}^{*}\geq 0,i=1,2,\cdots ,k \end{array}\right. \end{array}$$ (7)

where *ξ*_*i*_ and *ξ*_i_* are the relaxation variables, which represents the upper and lower limits of the training error under the error constraint (∣*y*_*i*_ − [*ω*^*T*^ ⋅ Φ(*x*) + *b*] ∣< *ε*). The error term *ε* is the maximum error allowed by the regression step, limits the number of support vector solutions and prevents over-generalization. Larger *ε* values imply less support vectors. The constant *C* > 0 controls the penalty for any sample that exceeds the error *ε*.

Expressions (6) and (7) represent a Convex Quadratic Optimization (CQO) problem. To solve the CQO problem, we express a Lagrange function

$$\begin{array}{cc} L & =\frac{1}{2}\omega^{T}\omega +C\sum_{i=1}^{k}\left(\xi_{i}+\xi_{i}^{*}\right)-\sum_{i=1}^{k}\alpha_{i}\left(\xi_{i}+\varepsilon -y_{i}+f\left(x_{i}\right)\right)\\ & -\sum_{i=1}^{k}\alpha_{i}^{*}\left(\xi_{i}^{*}+\varepsilon +y_{i}-f\left(x_{i}\right)\right)-\sum_{i=1}^{k}\left(\eta_{i}\xi_{i}+\eta_{i}^{*}\xi_{i}^{*}\right). \end{array}$$ (8)

where $\alpha_{i},\alpha_{i}^{*}\geq 0$, $\eta_{i},\eta_{i}^{*}\geq 0$ are the Lagrange multipliers. Then, we find the minimization of function *L* with respect to *ω*, *b*, *ξ*_*i*_ and $\xi_{i}^{*}$; the maximization of *L* with respect to $\alpha_{i},\alpha_{i}^{*},\eta_{i}$ and $\eta_{i}^{*}$; the maximization function of the dual form is obtained by substituting it into the Lagrange function:

$$\begin{array}{cc} \max_{\alpha_{i},\alpha_{i}^{*}}W\left(\alpha_{i},\alpha_{i}^{*}\right) & =-\frac{1}{2}\sum_{i,j=1}^{k}\left(\alpha_{i}-\alpha_{i}^{*}\right)\left(\alpha_{j}-\alpha_{j}^{*}\right)K\left(x_{i},x_{j}\right)\\ & -\varepsilon \sum_{i=1}^{k}\left(\alpha_{i}+\alpha_{i}^{*}\right)+\sum_{i=1}^{k}y_{i}\left(\alpha_{i}-\alpha_{i}^{*}\right). \end{array}$$ (9)

According to the KKT conditions, the following equations and constraints can be established

$$\begin{array}{c} \left\{ \begin{array}{cc} & \sum_{i=1}^{k}\left(\alpha_{i}-\alpha_{i}^{*}\right)=0\\ & 0\leq \alpha_{i},\alpha_{i}^{*}\leq C,i=1,2,\cdots ,k\\ & \omega =\sum_{i=1}^{k}\left(\alpha_{i}-\alpha_{i}^{*}\right)\Phi \left(x_{i}\right). \end{array}\right. \end{array}$$ (10)

where the kernel function *K*(*x*_*i*_, *x*_*j*_) = Φ(*x*_*i*_)^*T*^Φ(*x*_*j*_). This formulation describes the inner product of the high dimensional eigenspace. In this paper, we select the Radial Basis Kernel Function (RBKF) as a kernel function that is characterized by

$$\begin{array}{c} K\left(x,x_{i}\right)=exp\left(-\frac{\|x-x_{i}{\|}^{2}}{2\sigma^{2}}\right),\sigma >0. \end{array}$$ (11)

After solving, *α*_*i*_ and $\alpha_{i}^{*}$ are substituted into (10). Finally, the regression function is expressed by:

$$\begin{array}{c} f\left(x\right)=\sum_{i=1}^{k}\left(\alpha_{i}-\alpha_{i}^{*}\right)K\left(x,x_{i}\right)+b. \end{array}$$ (12)

### The influence of parameters

The performance of SVR is associated with the appropriate choice of parameter values. The parameter size has a considerable influence on SVR algorithm learning and generalization ability [50]. Therefore, determining the optimal support vector parameters is an important problem. The main parameters of the SVR model based on RBKF are penalty parameter *C* and kernel function parameter *σ*. The penalty parameter *C* is a trade-off parameter between the control error minimization and confidence interval maximization. The larger the *C*, the greater the penalty for the training error, which results in over-fitting; the smaller the *C*, the smaller the penalty for the empirical error, leading to a learning machine that is simpler but with higher (experience-based) risk. The kernel function parameter *σ* is related to the input space range and width of the learning sample; the larger the sample input space is, the greater the value. Conversely, the smaller the sample input space is, the smaller the value. Because the parameter search scope is sizable, and the parameter numbers are large, the optimal parameter is difficult to find. Thus, we optimized the parameters of SVR.

### Improved support vector regression

#### Support vector regression based on K-fold cross validation algorithm

Cross Validation (CV) is a random grouping of original data to some degree. One group is used as the test set and the others as the training set. Firstly, the training set is used to train the model, and the model is verified via the test set [51, 52]. The common CV methods are the Hold-Out Method, K-fold Cross Validation (K-CV) and Leave-One-Out Cross Validation (LOO-CV). In this paper, *C* and *σ* are optimized by the K-CV method under the training set. The SVR algorithm, after the K-CV parameter-optimization step is complete, is called K-CV-SVR. Its basic six steps are delineated in the pseudo-code that follows.

Step 1: Data preprocessing step. Reads in the sample set and preprocesses it.
Step 2: The sample set *S* is divided (equally) into *K* disjoint subsets, denoted as {*S*_1_, *S*_2_, ⋯, *S*_*K*_}.
Step 3: Take an element of the sample set (*S*_*i*_) as a test set without repetition. The remaining *K*−1 elements are used as the training set. The following subroutine is performed.
  for *C* = 2^−8^: 2^8^, *σ* = 2^−8^: 2^8^
  for *i* = 1: *K*
  Let *S*_*i*_ be the test sample *S*_*test*_
  Let *S*_1_ ⋃⋯⋃ *S*_*i*−1_ ⋃ *S*_*i*+1_ ⋃⋯⋃ *S*_*K*_ be the training sample *S*_*train*_
  Use *S*_*train*_ to train SVR
  Input the *S*_*test*_ into the trained SVR model to determine the test error
Step 4: After the previous step, for each parameter pair {*C*, *σ*}, the *K* test errors and *K* models can be obtained. The average of the squared error sum of the *K* model test set is called the MSE.
Step 5: When all possible parameter {*C*, *σ*} are traversed, we select the parameters that minimize the MSE to be the optimal parameter {*C*^*best*^, *σ*^*best*^}.
Step 6: Using the training sample set, the SVR model with parameters {*C*^*best*^, *σ*^*best*^} is established, and the network is trained to complete the prediction.

**Fig 1:**
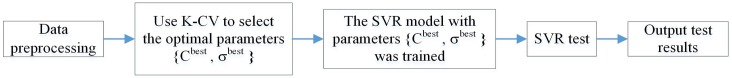
Flow chart of SVR algorithm based on K-CV.

The specific process of the GA-SVR algorithm is shown in Fig 1.

#### Support vector regression based on genetic algorithm

The Genetic Algorithms (GA) were proposed and developed by professor Holland of the University of Michigan in 1962. It is a search algorithm that is based on the biological evolutionary process of the survival of the fittest in nature [53]. The basic process is as follows: First, we randomly generate a population of a certain size for the problem to be solved, the adaptive value of each individual is calculated and the fitness assessment was performed for all individuals in the group. Second, by selecting, crossing, and mutating a group of individuals, a set of individuals more adaptable to the environment is produced. Finally, based on the new generation, the three operations that select, cross and mutate are carried out. After several generations of evolution, until the set termination conditions are satisfied, the optimal solution to the problem is found [54]. The nine basic steps of the genetic algorithm applied to SVR parameter optimization (GA-SVR) are delineated in the steps that follow.

Step 1: Data preprocessing. To avoid a large magnitude difference between the various factors, the input sample is normalized.
Step 2: Initialization of the population. The penalty parameter *C* and the kernel function parameter *σ* are initialized and binary coding is performed. The initial population *P*(*t*) of the genetic algorithm is constructed. The parameters of the GA are set, such as the initial population size of the GA, the maximum genetic algebra *T*, crossover rate and mutation rate. A set of chromosomes that represent the SVR parameter values are randomly generated and each chromosome is composed of {*C*, *σ*}.
Step 3: Individual evaluation. Regression training was performed for each individual generated by the population. The reciprocal of the mean square error (found during training) is used as the objective function value, i.e., the individual fitness. The fitness function value of each individual is calculated, and the calculation formula is:
  $$\begin{array}{c} F=\frac{1}{m}\sum_{i=1}^{m}{\left(y_{i}-\bar{y_{i}}\right)}^{2} \end{array}$$ (13)
  where *m* is the number of network output nodes, *y*_*i*_ is the expected output of the *i*-th node of the SVR and $\bar{y_{i}}$ is the prediction output of the *i*-th node.
Step 4: Select operation. Individual fitness is based on roulette method, the chromosomes with higher fitness values were selected from the current population to replicate.
Step 5: Cross operation. Two individuals in the population were selected as the parent body, a new generation of chromosomes were obtained via a cross operation associated with some probability. A single point-cross method is used here.
Step 6: Mutation operation. Randomly select individuals in a population and change some genes in individuals with certain probabilities. This results in a new set of individuals.
Step 7: Termination conditions. If *t* ≤ *T*, then go to step. If *t* > *T* or if the optimal individual continues to be less than a constant, then the algorithm is automatically terminated and the output is the optimal value.
Step 8: Optimal decoding. This step outputs the optimal parameter {*C*^*best*^, *σ*^*best*^} of the SVR.
Step 9: Using the training set, the SVR model with parameters {*C*^*best*^, *σ*^*best*^} are established. The network is now trained and the test sample is placed back into the training set. Thus, SVR achieves the predicted results.

**Fig 2:**
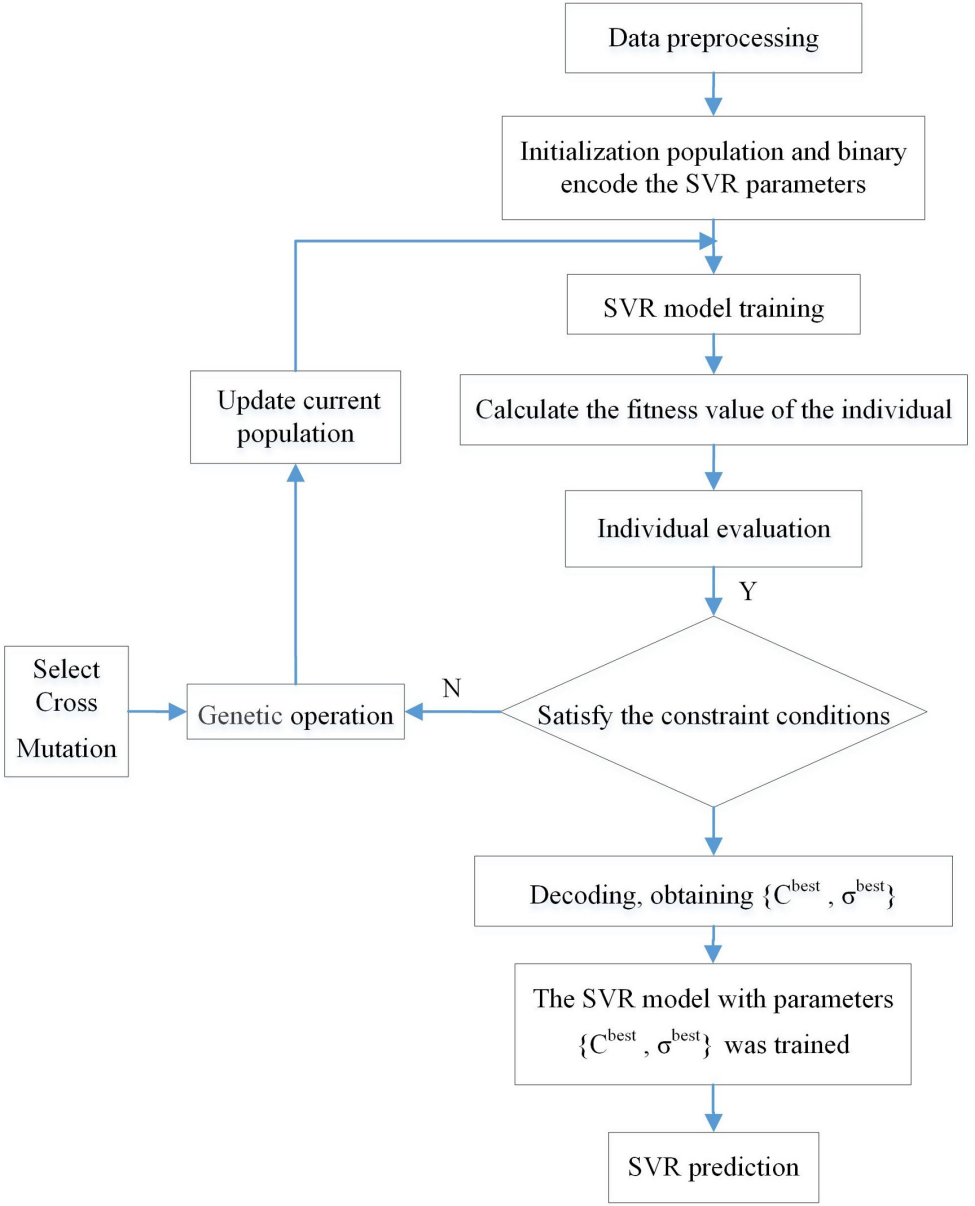
SVR parameter optimization based on GA.

The specific process of the GA-SVR algorithm is shown in Fig 2.

#### Support vector regression based on improved particle swarm optimization

Particle Swarm Optimization (PSO) algorithm is an optimization algorithm based on swarm intelligence theory, which was first proposed by Kennedy and Eberhart in 1995 [55]. It is a global optimization algorithm that simulates bird-predator behavior and achieves the goal of optimization via a collective cooperation among birds. The solution of each optimization problem in the PSO is considered a bird in the search space called the “particle”. Each particle is associated with its own position and speed, which is dynamically adjusted according to its “flight experience” and the influence of other particles in the set [56, 57]. The PSO is an optimal solution search method for a particle that follows the current optimal method to find (determine) a particle in the solution space. To measure the superiority of each particle solution, a fitness value function is defined. In this paper, the mean square error is taken as the fitness function, which can directly reflect the performance of SVR regression.

The basic principle of the PSO algorithm is described using mathematical terminology. Suppose that in an *n*-dimensional solution space, the population *X* = (*X*_1_, *X*_2_, ⋯, *X*_*m*_) is composed of *m* particles, where *X*_*i*_ = (*x*_*i*1_, *x*_*i*2_, ⋯, *x*_*in*_) is the current coordinate position of the *i*-th particle, *V*_*i*_ = (*v*_*i*1_, *v*_*i*2_, ⋯, *v*_*in*_) is the current velocity of the *i*-th particle, *P*_*ibest*_ is the current best position of *i*-th particle and *P*_*gbest*_ is the best location of the whole particle swarm. According to the basic principle of the optimal particle, each particle *X*_*i*_, *i* = 1, 2, ⋯, *m* will update its velocity and position according to velocity adjustment Eq (14) and position adjustment Eq (15).

$$\begin{array}{c} \begin{array}{c} V_{i}\left(t+1\right)=\omega V_{i}\left(t\right)+c_{1}r_{1}\left(P_{ibest}-X_{i}\left(t\right)\right)+c_{2}r_{2}\left(P_{gbest}-X_{i}\left(t\right)\right). \end{array} \end{array}$$ (14)

$$\begin{array}{c} X_{i}\left(t+1\right)=X_{i}\left(t\right)+V_{i}\left(t+1\right). \end{array}$$ (15)

where *ω* is the inertia weight, *t* is the current evolutionary algebra, *r*_1_ and *r*_2_ are random numbers distributed between [0, 1]; *c*_1_ and *c*_2_ are the accelerated constants that are usually evaluated between (0, 2]; *V*_*i*_(*t*) is the original velocity of the particle and *V*_*i*_(*t* + 1) is the new velocity of the particle.

To reduce the possibility of particles leaving the search space during evolution, the flight velocity *V*_*i*_ of a particle is usually limited to a certain range, namely *V*_*i*_ ∈ [−*V*_*max*_, *V*_*max*_].

$$\begin{array}{c} \left\{ \begin{array}{cc} V_{i}=V_{max},\; & if\;V_{i}>V_{max}\\ V_{i}=-V_{max},\; & if\;V_{i}<-V_{max}. \end{array}\right. \end{array}$$ (16)

The adjustment of equation *P*_*ibest*_ and *P*_*gbest*_ are show in Eqs (17) and (18), where *f*(*x*) is the fitness function.

$$\begin{array}{c} P_{ibest}\left(t+1\right)=\{\begin{array}{ccc} & P_{ibest}\; & if\;f\left(X_{i}\left(t+1\right)\right)\geq f(P_{ibest})\\ & X_{i}\left(t+1\right)\; & if\;f\left(X_{i}\left(t+1\right)\right)<f(P_{ibest}). \end{array} \end{array}$$ (17)

$$\begin{array}{c} \begin{array}{cc} & \{P_{gbest}\left(t\right)\in \left\{P_{1best}\left(t\right),\cdots ,P_{mbest}\left(t\right)\right\}|f\left(P_{gbest}\left(t\right)\right)=\\ & min\{f\left(P_{1best}\left(t\right)\right),\cdots ,f\left(P_{mbest}\left(t\right)\right)\}\}. \end{array} \end{array}$$ (18)

$$\begin{array}{c} f\left(X_{i}\right)=\frac{1}{m}\sum_{i=1}^{m}{\left(y_{i}-\bar{y_{i}}\right)}^{2}. \end{array}$$ (19)

where *m* is the number of particles, *y*_*i*_ is the actual value and $\bar{y_{i}}$ is the predictive value.

The inertia weight *ω* is mainly used to balance the global search capability and local development capability of particles in Eq (14). A larger inertia weight results in overly rapid particle velocity and deviation from the search area of the optimal solution. A smaller inertia weight gives the particle stronger local search ability, but takes a longer time to find the global optimal solution. Therefore, careful selection of inertia weight is important to obtain good performance. In this paper, the inertia weight *ω* is taken as

$$\begin{array}{c} \omega \left(t\right)=\omega_{max}\cdot (\frac{\omega_{min}}{\omega_{max}}{)}^{1+\frac{t}{T_{max}}}. \end{array}$$ (20)

where *ω*_*max*_ is the maximum inertia weight; *ω*_*min*_ is the minimum inertia weight; *t* is the current iteration number; *T*_*max*_ is the maximum iteration number. In order to be distinguished from PSO, PSO with the inertia weight (20) is named as IPSO.

The algorithm for the IPSO-optimized SVR parameter is called IPSO-SVR and its basic steps are as follows

Step 1: Data preprocessing. Read in the sample set and preprocess it.
Step 2: PSO initialization. A particle is composed of a penalty parameter *C* and a kernel function parameter *σ*. We initialize the particle swarm {*C*, *σ*}, determine the population size of PSO, randomly generate the initial position and velocity of the particle, set the maximum number iterations *T*_*max*_ of the algorithm and the range of velocity [−*V*_*max*_, *V*_*max*_].
Step 3: We calculate the fitness function value of each particle. The prediction error of the current position of each particle is obtained by using the SVR corresponding to each particle vector to predict the learning sample. The fitness function value of each particle is calculated by using Eq (19).
Step 4: Update the personal best *P*_*ibest*_ of particle. The fitness of each particle is evaluated. If the fitness value of the current iteration is better than the personal best *P*_*ibest*_, then *P*_*ibest*_ is replaced by the current fitness value; otherwise, the original value is retained.
Step 5: We update the global best of the population. If the fitness value of a particle is better than the current global best *P*_*gbest*_, then *P*_*gbest*_ is updated; otherwise, the original value is retained.
Step 6: We update the velocity, position and inertia weight of the particle according to Eqs (14), (15), and (20).
Step 7: Termination conditions. Either the current number of iterations *t* = *T*_*max*_ or fitness values is less than (the provided) precision *ε*. If *ε* is satisfied, then the optimization step is complete, the optimal parameter {*C*^*best*^, *σ*^*best*^} is obtained and the algorithm moves on to step 8. Otherwise, let *t* = *t* + 1, and the algorithm returns to step 3.
Step 8: We implement the training sample set, the SVR model with parameters {*C*^*best*^, *σ*^*best*^} are determined. The network is trained and the test sample is placed back into the trained SVR to get the predicted results.

**Fig 3:**
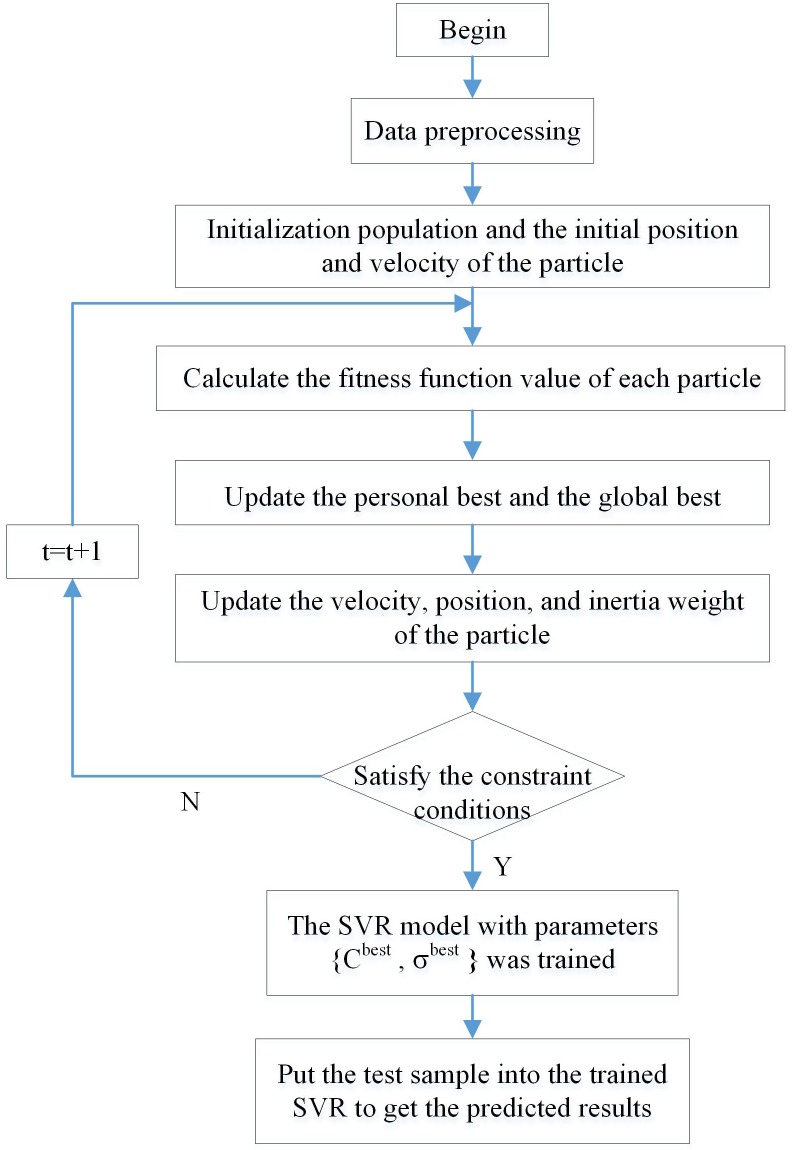
The SVR algorithm based on IPSO.

The specific process of the IPSO-SVR algorithm is shown in Fig 3.

## Experimental

### The data source

Influenza-like cases are a main indicator for the condition monitoring of flu, both domestically and overseas. These cases refer to a specific set of symptoms provided by specific outpatient cases of sentinel hospitals. The symptoms are fever with a temperature equal to or greater than 38 °C, a cough or a sore throat. These cases often lack diagnosis based on laboratory findings. The source of flu data in this article mainly consists of official data from the 10 regional flu outbreaks in the US and the twitter data for the same period. In [58], the 10 regions defined by Health and Human Services (HHS) can be easily identified. The software used to simulate the prediction model is MATLAB (R2014a). The official data used in this study was acquired from the historical ILI weekly (https://gis.cdc.gov/grasp/fluview/fluportaldashboard.html) data set which was published by the Center for Disease Control and Prevention S1 Table. Twitter data refers to data in [33], it is derived from flu data provided by the prototype of flu-surveillance system that was established by Wang et al. [26]. In this paper, we collected 55 weeks of data in 10 regions of the US from the 41st week of 2016 to the 43rd week of 2017. ILI data from the 41st week of 2016 to the 38th week of 2017 was selected as the training set to use for model building. ILI data from the 39th week to 43rd week of 2017 was selected as the test set to use for model validation.

### The data processing

The vectors in the original data sample use various orders of magnitude and the order of magnitude varies from sample to sample. To avoid the outliers based on data ranges, the data was normalized. The Mapminmax MATLAB function was used to normalize the sample to be constrained within the [0, 1] interval. The disadvantage of dimensional inconsistency to model was eliminated and the operation efficiency of the model was improved. The normalized formula is

$$\begin{array}{c} X_{t}^{\prime }=\frac{X_{t}-X_{min}}{X_{max}-X_{min}}. \end{array}$$ (21)

where $X_{t}^{\prime }$ is the normalized data, *X*_*t*_ is the original input data and *X*_*max*_,*X*_*min*_ are the largest and smallest value in the historical data, respectively.

After predicting the output, a reverse normalization process is performed and the actual predicted ILI were obtained. The reverse normalization formula is

$$\begin{array}{c} X_{t}=\left(X_{max}-X_{min}\right)X_{t}^{\prime }+X_{min}. \end{array}$$ (22)

### Experimental results and analysis

**Fig 4:**
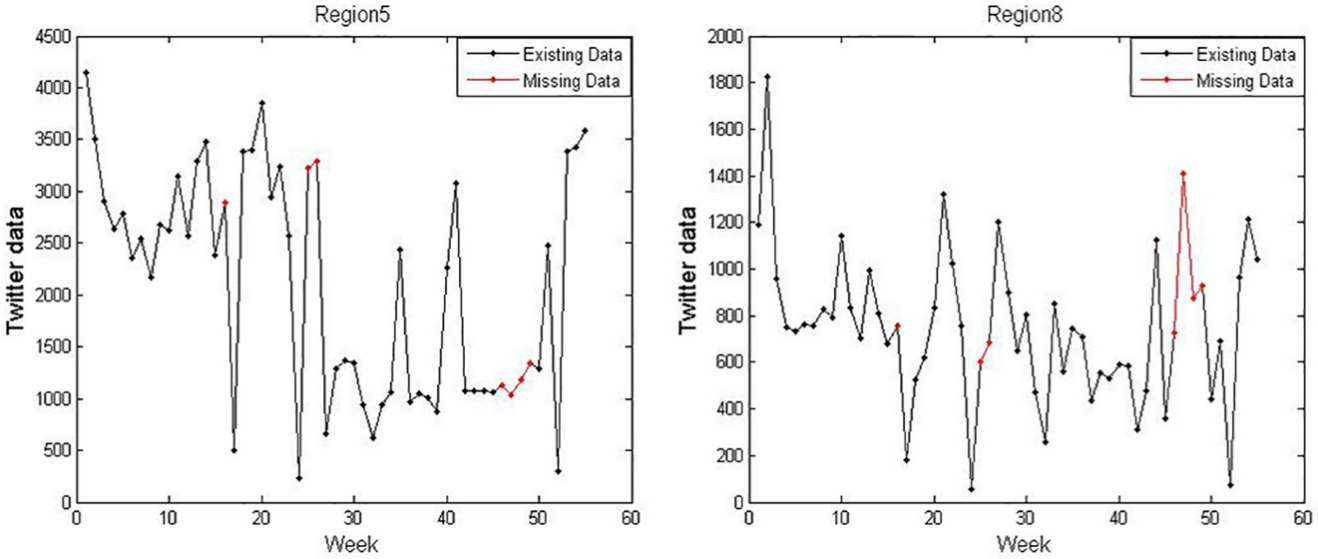
The twitter data of regions 5 and 8.

In this study, the twitter data for regions 1-10 misses the 16th, 25th-26th, and 46th-49th data. We use the SVR for prediction to revise these missing data. We perform twenty times and take the corresponding prediction of the missing data with the minimum MAPE. For example, the twitter data of regions 5 and 8 are shown in Fig 4. The red dots in Fig 4 represent our predictions.

To evaluate the prediction performance of the various prediction models, each model was evaluated by the Mean Square Error (MSE), Relative-Mean-Square Error (RMSE), Mean Absolute Percentage Error (MAPE) and calculation time.

$$\begin{array}{c} MSE=\frac{1}{n}\sum_{i=1}^{n}{\left(y_{i}-\bar{y_{i}}\right)}^{2}. \end{array}$$ (23)

$$\begin{array}{c} MAPE=\frac{1}{n}\sum_{i=1}^{n}|\frac{y_{i}-\bar{y_{i}}}{\bar{y_{i}}}|\times 100\%. \end{array}$$ (24)

$$\begin{array}{c} RMSE=\frac{1}{n}\sum_{i=1}^{n}{\left(\frac{y_{i}-\bar{y_{i}}}{\bar{y_{i}}}\right)}^{2}. \end{array}$$ (25)

In these evaluation methods, *n* is the number of samples; *y*_*t*_ is the actual value of period *t*; and $\bar{y_{t}}$ is the predicted value of period *t*.

In this work, we predict %ILI using CV-SVR, GA-SVR, PSO-SVR, and IPSO-SVR methods. The independent variable of models 1-3 were used as feature inputs of these ML methods and the dependent variable was used as the output. We compared the prediction results generated by the IPSO-SVR with CV-SVR, GA-SVR, and PSO-SVR methods (which are based on models 1-3). The prediction results are shown in Tables 1–3, and calculation times are shown in Table 4.

**Table 1:** Evaluation index results of model 1 for 10 regions of US.

Region	Error	CV-SVR	GA-SVR	PSO-SVR	IPSO-SVR
1	MSE	1.2247	1.1309	0.8836	0.8075
	RMSE	1.7433	1.6070	1.3214	1.2458
	MAPE	1.0936	1.0626	1.0069	0.8252
2	MSE	0.5515	0.7651	0.5810	0.5447
	RMSE	0.1952	0.2730	0.1904	0.1794
	MAPE	0.3949	0.4827	0.3181	0.3215
3	MSE	2.7369	2.7564	0.7368	0.7378
	RMSE	1.8162	1.8549	0.5134	0.5139
	MAPE	1.1778	1.1505	0.5987	0.5989
4	MSE	7.2071	9.4694	11.1045	11.7234
	RMSE	2.7272	3.5422	4.2457	4.6025
	MAPE	1.5791	1.8344	2.0160	2.0881
5	MSE	1.6050	1.7586	0.2454	0.2272
	RMSE	1.2276	1.3436	0.1861	0.1724
	MAPE	0.9989	1.0861	0.3537	0.3425
6	MSE	0.0759	2.1083	0.0638	0.0582
	RMSE	0.0172	0.5241	0.0132	0.0118
	MAPE	0.1164	0.6709	0.0983	0.0883
7	MSE	2.4362	2.5517	2.0060	1.6671
	RMSE	2.4432	2.4943	2.0476	1.6982
	MAPE	1.4827	1.4981	1.3379	1.2257
8	MSE	0.2397	0.2608	0.1347	0.1330
	RMSE	1.0788	1.0961	0.5915	0.5821
	MAPE	0.8556	0.8319	0.5967	0.5833
9	MSE	0.0367	0.0806	0.0249	0.0248
	RMSE	0.0147	0.0332	0.0100	0.0099
	MAPE	0.1041	0.1609	0.0814	0.0812
10	MSE	0.8944	0.8979	0.8503	0.8401
	RMSE	0.8648	0.8689	0.8350	0.8225
	MAPE	0.7786	0.7806	0.7175	0.7082

**Table 2:** Evaluation index results of model 2 for 10 regions of US.

Region	Error	CV-SVR	GA-SVR	PSO-SVR	IPSO-SVR
1	MSE	0.3803	0.3811	0.5575	0.3612
	RMSE	0.6677	0.6683	0.9634	0.6356
	MAPE	0.6940	0.6884	0.8843	0.6841
2	MSE	1.4038	0.8164	2.0968	1.9606
	RMSE	0.5899	0.3746	1.0149	0.8310
	MAPE	0.6131	0.5070	0.7272	0.7451
3	MSE	1.8137	1.7965	1.6740	1.6977
	RMSE	1.3737	1.3615	1.2818	1.3016
	MAPE	0.9777	0.9760	0.9518	0.9526
4	MSE	1.8745	1.9831	1.7528	1.3208
	RMSE	0.6592	0.7418	0.6787	0.4623
	MAPE	0.7639	0.7279	0.7023	0.5795
5	MSE	0.8477	0.8379	1.1876	0.8092
	RMSE	0.6482	0.6413	0.9080	0.6207
	MAPE	0.7482	0.7528	0.9035	0.7327
6	MSE	0.8563	0.8644	0.8550	0.8459
	RMSE	0.2144	0.2213	0.2128	0.1884
	MAPE	0.4013	0.3915	0.4032	0.3781
7	MSE	0.4899	0.4960	0.6058	0.4313
	RMSE	0.5341	0.5409	0.5221	0.4841
	MAPE	0.6587	0.6853	0.6517	0.6073
8	MSE	0.7583	0.3963	0.7538	0.6458
	RMSE	2.1670	1.1701	2.3610	1.7764
	MAPE	1.1702	0.8843	1.1066	1.0388
9	MSE	0.2956	0.1221	0.3401	0.3395
	RMSE	0.1200	0.0588	0.1387	0.1369
	MAPE	0.2853	0.2198	0.3271	0.3035
10	MSE	0.1334	0.1097	0.7560	0.2935
	RMSE	0.1074	0.1554	0.8328	0.3150
	MAPE	0.2836	0.3134	0.6261	0.5104

**Table 3:** Evaluation index results of model 3 for 10 regions of US.

Region	Error	CV-SVR	GA-SVR	PSO-SVR	IPSO-SVR
1	MSE	0.0127	0.0217	0.0211	0.0123
	RMSE	0.0225	0.0419	0.0388	0.0221
	MAPE	0.1329	0.1880	0.1659	0.1203
2	MSE	0.0125	0.0132	0.0126	0.0111
	RMSE	0.0044	0.0051	0.0046	0.0042
	MAPE	0.0596	0.0617	0.0580	0.0575
3	MSE	0.0285	0.0279	0.0238	0.0163
	RMSE	0.0225	0.0220	0.0189	0.0130
	MAPE	0.1489	0.1473	0.1361	0.1124
4	MSE	0.0314	0.0355	0.0315	0.0205
	RMSE	0.0098	0.0111	0.0098	0.0076
	MAPE	0.0795	0.0797	0.0726	0.0724
5	MSE	0.0106	0.0108	0.0086	0.0082
	RMSE	0.0086	0.0087	0.0068	0.0065
	MAPE	0.0666	0.0664	0.0603	0.0583
6	MSE	0.0212	0.0204	0.0225	0.0237
	RMSE	0.0050	0.0049	0.0055	0.0058
	MAPE	0.0578	0.0577	0.0610	0.0622
7	MSE	0.0143	0.0270	0.0598	0.0598
	RMSE	0.0200	0.0314	0.0645	0.0645
	MAPE	0.0979	0.1444	0.1969	0.1971
8	MSE	0.0381	0.0314	0.0523	0.0248
	RMSE	0.0692	0.0562	0.0976	0.0458
	MAPE	0.2460	0.2177	0.2910	0.1893
9	MSE	0.0498	0.0504	0.0472	0.0426
	RMSE	0.0194	0.0199	0.0185	0.0164
	MAPE	0.1159	0.1226	0.1155	0.0987
10	MSE	0.0433	0.0432	0.0464	0.0421
	RMSE	0.0485	0.0484	0.0508	0.0406
	MAPE	0.1495	0.1697	0.1596	0.1463

**Table 4:** Comparison of the calculation time for each algorithm (in models 1-3).

Model	CV-SVR	GA-SVR	PSO-SVR	IPSO-SVR
Model 1	242s	31s	25s	20s
Model 2	244s	32s	27s	26s
Model 3	213s	54s	26s	25s

**Fig 5:**
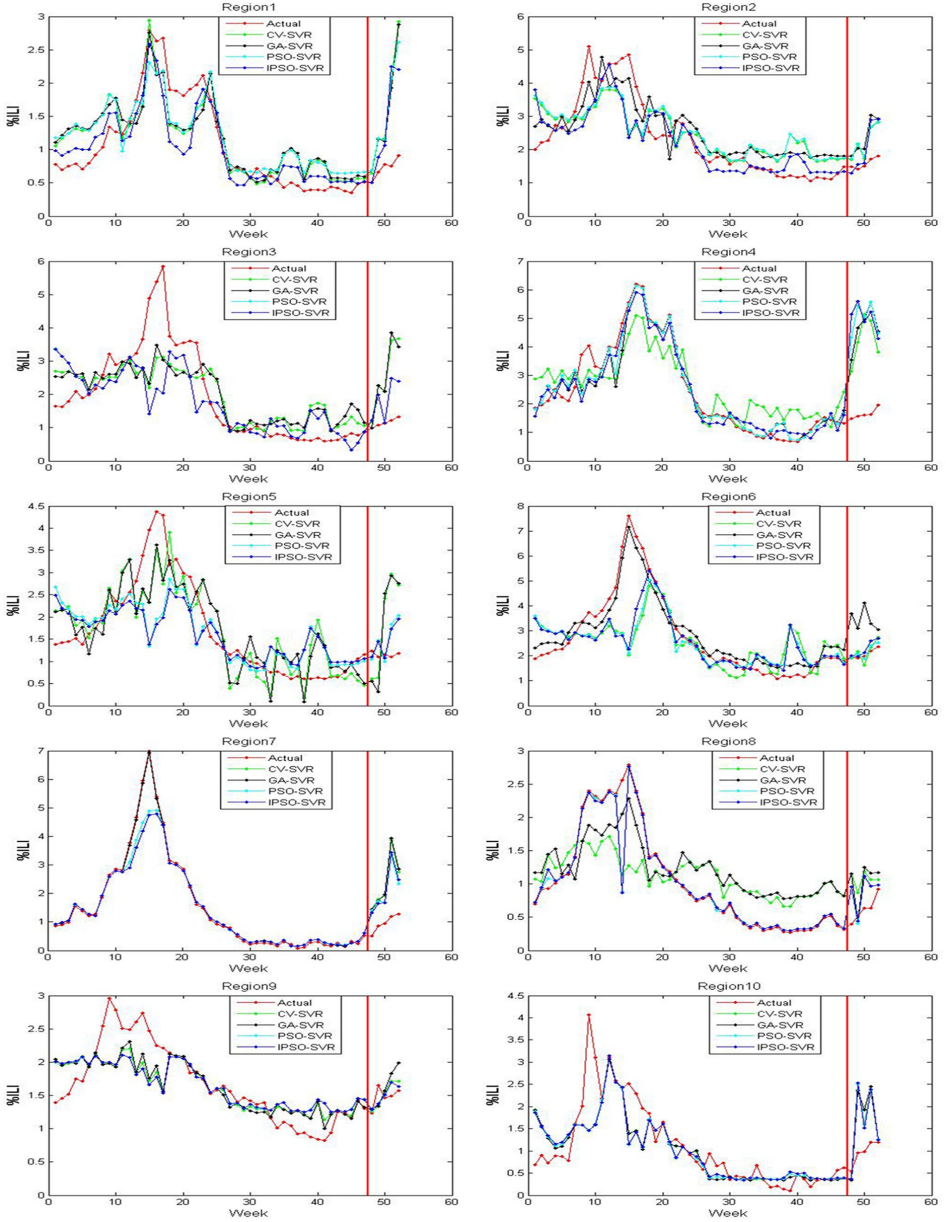
The trained and tested results of model 1 for 10 regions.

**Fig 6:**
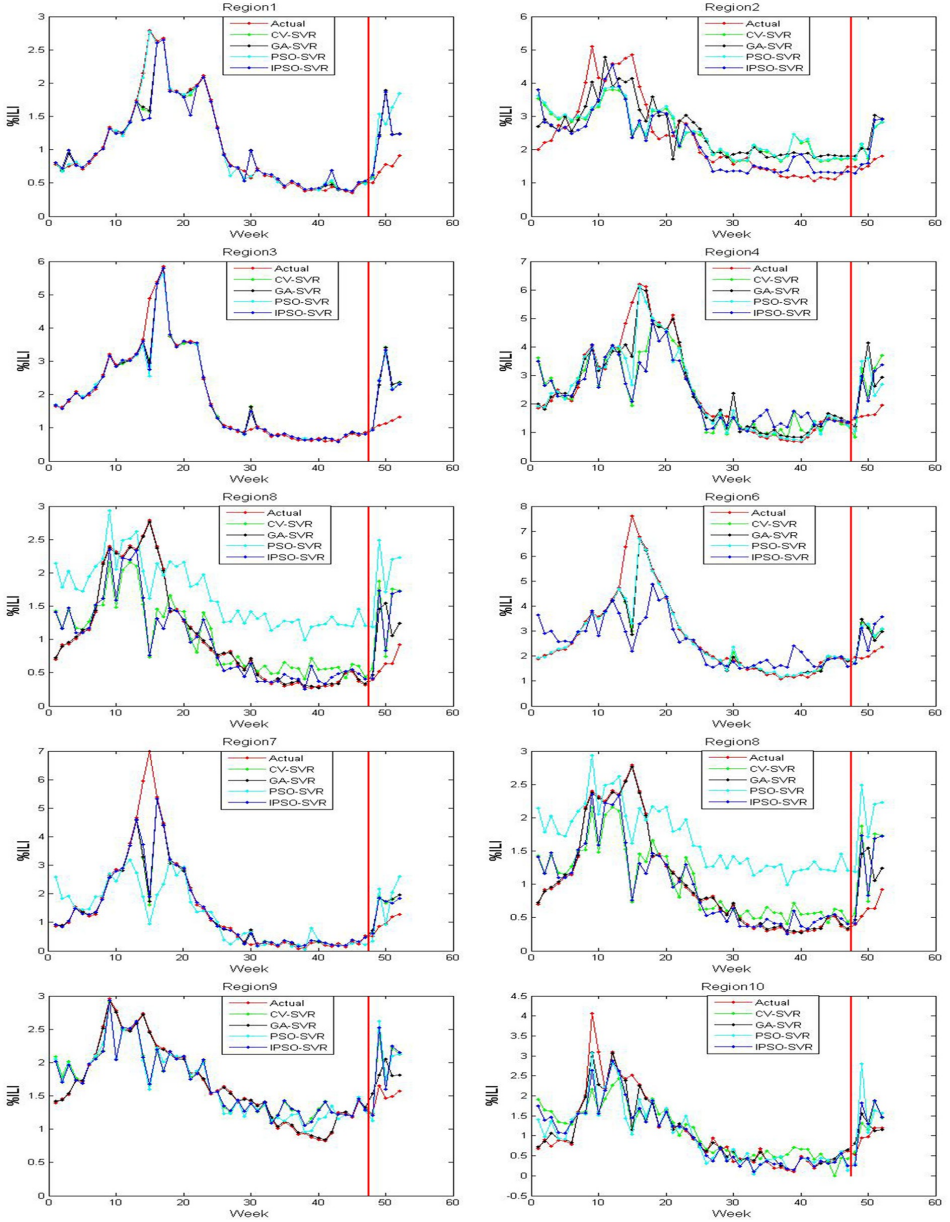
The trained and tested results of model 2 for 10 regions.

**Fig 7:**
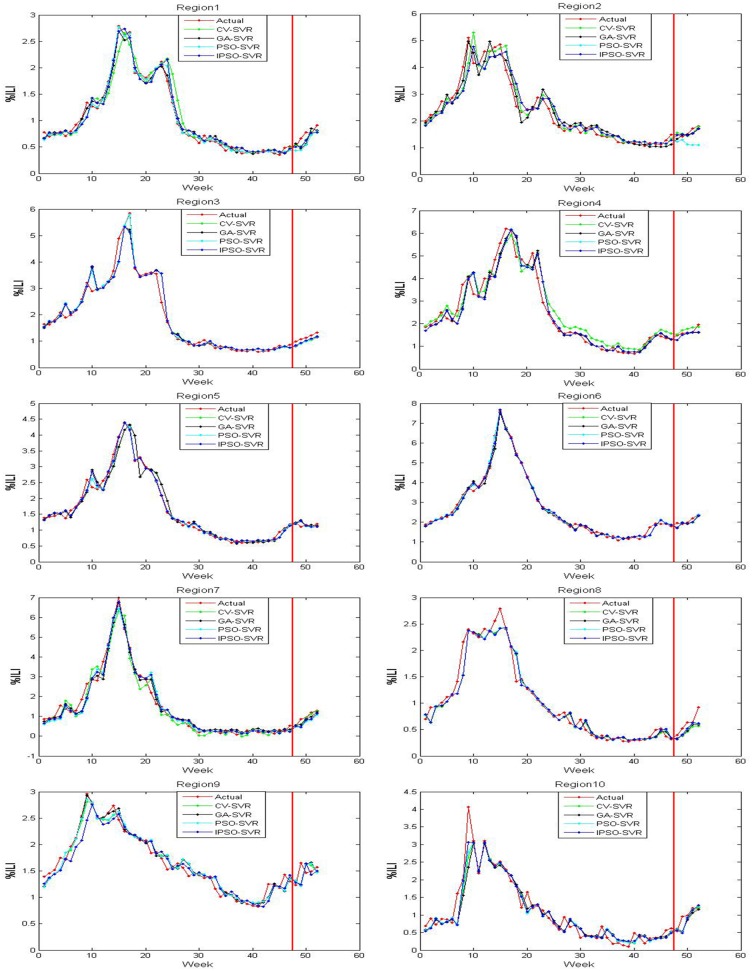
The trained and tested results of model 3 for 10 regions.

The actual value and predicted value of models 1-3 on the trained and tested samples of ten regions are shown in Figs 5–7, where the red line perpendicular to the horizontal axis in every subfigure divides the whole plate into two parts: the left part is the actual outputs and predicted outputs of the training samples on models 1-3, and right part is the actual outputs and the predicted outputs of the test samples on models 1-3. From Figs 5–7, we can see that the output of model 3 are the closest to the actual output in the training samples and test samples. The output of model 2 are close to the actual output in the training samples, but there are differences between the predicted output of model 2 and the actual output in the test samples. There are many discrepancies between the estimates using model 1 and the actual CDC values in the training samples and test samples.

Tables 1–3 shows the MSE, RMSE, and MAPE on the test samples of ten regions for models 1-3, respectively. By comparing the MSE, RMSE, and MAPE results of models 1 and 2, we find that model 2 has higher ILI prediction capabilities for most regions; this is because the MSE, RMSE and MAPE values are smaller. These results suggest that the flu epidemic other regions has an impact on the current region, which means that the spread of flu is also affected by an inter-regional spread of flu. Therefore, the %ILI prediction should not only consider the role of the current regional flu data, but should also consider the impact of flu transmission in extended regional areas. Similarly, compared to the MSE, RMSE, and MAPE results of model 2 with model 3, we found that the prediction effect of the model 3 was better than the model 2 over all 10 regions. The results show that the twitter data complements with CDC ILI data, that is, the twitter data may contain new information that CDC data does not have. This information reflects the variation trend of the previous period of the flu that typically lasts 1-2 weeks.

By comparing the IPSO-SVR, PSO-SVR, GA-SVR, and CV-SVR prediction results of model 1, we find that the prediction effect of the IPSO-SVR, in most regions (1-2 and 5-9) showed more robustness than the other three methods. By comparing the IPSO-SVR, PSO-SVR, GA-SVR, and CV-SVR prediction results of model 2, we find that the prediction effect of the IPSO-SVR was better than other three methods in most regions (1 and 4-7). By comparing the IPSO-SVR, PSO-SVR, GA-SVR, and CV-SVR prediction results of model 3, we find that the prediction effect of the IPSO-SVR, in most regions (1-5 and 8-9) showed more robustness than the other three methods. By comparing the IPSO-SVR, PSO-SVR, GA-SVR and CV-SVR, the calculation time to find a result (based on models 1-3), we find that IPSO-SVR has the shortest calculation time than the other three algorithms.

Several comparison can be made to illustrate that the IPSO-SVR prediction results of model 3 are the best of the three models since its MSE, RMSE and MAPE values are the smallest, and the run-time of the IPSO-SVR method is the shortest. Meanwhile, the prediction results were compared to the results of the BP neural network influenza prediction model based on improved artificial tree algorithm in reference [33]. We can show that the IPSO-SVR prediction results of model 3 are better than the prediction results of the IAT-BPNN model in most regions (1-3, 5-6, and 8-10) under review. The precision is higher, better and more reflects the ground truth of flu transmission. Results show that in flu prediction, the IPSO-SVR algorithm can predict %ILI more effectively, while showing that the method of using IPSO to optimize the SVR parameters is feasible and effective, and that the techniques not only provide new methods to further the development of flu prediction, but also have important reference value for the further applications of SVR.

## Conclusions

In this paper, we proposed three flu prediction models that use US-based twitter and CDC data. Then, we proposed an improved PSO to optimize the parameters of SVR. The independent and dependent variables of models 1-3 are used as input and output of the IPSO-SVR for predicting the CDC’ unweighted %ILI of US. Comparing the prediction results of IPSO-SVR, PSO-SVR, GA-SVR, and CV-SVR for models 1-3. The experimental results show that 1) flu outbreaks in adjacent areas also have an impact on the current spread of flu in a region; 2) the twitter data complements with CDC ILI data; 3) the IPSO-SVR prediction results of model 3 was better than the prediction results of IAT-BPNN model; 4) the IPSO-SVR prediction results of model 3 for %ILI are not only suitable for ten regions defined by HHS, but also generates an optimization algorithm that can be applied to optimize the SVR parameters, which used to solve the other predict problem.

## Supporting information

S1 Table CDC data. Distribution of ILIs from week 41^*st*^ in 2016 to week 43^*rd*^ in 2017 in ten regions of the United States.
(XLSX)
